# Determination of ACC-induced cell-programmed death in roots of *Vicia faba* ssp. *minor* seedlings by acridine orange and ethidium bromide staining

**DOI:** 10.1007/s00709-012-0383-9

**Published:** 2012-02-15

**Authors:** Anna Byczkowska, Anita Kunikowska, Andrzej Kaźmierczak

**Affiliations:** Department of Cytophysiology, University of Łódź, Pomorska 141/143, PL-90236 Łódź, Poland

**Keywords:** Aerenchyma, Cell membrane integrity, Ethylene, Fluorescence, Programmed cell death, Root cortex cells

## Abstract

Fluorescence staining with acridine orange (AO) and ethidium bromide (EB) showed that nuclei of cortex root cells of 1-aminocyclopropane-1-carboxylic acid (ACC)-treated *Vicia faba* ssp. *minor* seedlings differed in color. Measurement of resultant fluorescence intensity (RFI) showed that it increased when the color of nuclear chromatin was changed from green to red, indicating that EB moved to the nuclei via the cell membrane which lost its integrity and stained nuclei red. AO/EB staining showed that changes in color of the nuclear chromatin were accompanied by DNA condensation, nuclei fragmentation, and chromatin degradation which were also shown after 4,6-diamidino-2-phenylindol staining. These results indicate that ACC induced programmed cell death. The increasing values of RFI together with the corresponding morphological changes of nuclear chromatin were the basis to prepare the standard curve; cells with green unchanged nuclear chromatin were alive while those with dark orange and bright red nuclei were dead. The cells with nuclei with green–yellow, yellow–orange, and bright orange chromatin with or without their condensation and fragmentation chromatin were dying. The prepared curve has became the basis to draw up the digital method for detection and determination of the number of living, dying, and dead cells in an *in planta* system and revealed that ACC induced death in about 20% of root cortex cells. This process was accompanied by increase in ion leakage, shortening of cells and whole roots, as well as by increase in weight and width of the apical part of roots and appearance of few aerenchymatic spaces while not by internucleosomal DNA degradation.

## Introduction

Programmed cell death (PCD) is an active process controlling proper development of unicellular as well as multicellular organisms (van Doorn and Woltering 2005) by elimination of physiologically redundant, damaged, or abnormal cells (Cacas 2010; Taraphdar et al. 2001).

In animals, PCD may take the form of apoptosis, micro- and macroautophagy, or non-lysosomal (van Doorn and Woltering 2005) as well as mitotic catastrophe, in which cells can die via apoptosis or necrosis (Hübner et al. 2009). The latest classification described by van Doorn et al. (2011) rightly confirms that apoptosis does not exist in plants (van Doorn and Woltering 2005), and death-dependent development of plants, ultrastructurally defined, is controlled via vacuolar, necrotic, as well as mixed types of plant cell death (van Doorn et al. 2011). Vacuolar cell death is connected with the formation of lytic vacuoles, which increase in volume; gradual decrease of the cytoplasm; formation of actin cables; nuclear envelope disassembly; as well as nuclei segmentation and chromatin condensation (van Doorn 2011; van Doorn et al. 2011). Necrotic plant cell death is defined by the absence of growing lytic vacuoles, swelling of mitochondria, early rupture of plasma membrane, and shrinkage of the protoplast. The third type of plant cell death is connected with hypersensitive response, characterized by a variety of morphological hallmarks (van Doorn et al. 2011).

However, attempted classification of cell death using metabolic (biochemical and cell biological) markers of PCD is not so obvious because frequently in different combinations, during many procedures, obtained results although allowing to give the most multilevel picture of this process, with many similar features of cell death between plants and animals (Jan et al. 2008), do not allow to indicate the type of death (Cacas 2010; Collazo et al. 2006; Jan et al. 2008; Kwon and Park 2008), suggesting that features of death are closely connected with the type of cells.

Detection and evaluation are the most important steps of cell death studies. The following methods are used to study cell death both in plants and animals: (1) analyses of cell viability with 3-(4,5-dimethylthiazol-2-yl)-2,5-diphenyltetrazolium bromide or trypan blue (Raju et al. 2004), (2) determination of free 3′OH ends in the nuclear chromatin with the TUNEL method (terminal deoxynucleotidyl transferase mediated dUTP Nick End Labeling; Kobori et al. 2007; Palavan-Unsal et al. 2005), (3) detection of internucleosomal fragmentation of DNA with agarose gel eletrophoresis (Kobori et al. 2007; Palavan-Unsal et al. 2005) and commet assay (Cabello et al. 2009), and (4) measurement of loss of nuclear DNA (Palavan-Unsal et al. 2005) as well as (5) assessment of mitochondrial transmembrane potential and intracellular reactive oxygen species (Ishii et al. 2002), (6) measurement of an ion leakage (Palavan-Unsal et al. 2005), (7) detection of cytochrome c release (Cacas 2010; Kawai-Yamada et al. 2004; Mlejnek and Doležel 2005), (8) analyses of annexin V, (9) determination of the activity of specific proteases, caspases (caspase-3/7; Ishii et al. 2002; Mlejnek and Doležel 2005; Palavan-Unsal et al. 2005), (10) detection of anti- and proapoptotic (Bax and Bcl-2, respectively) proteins (Cabello et al. 2009; Kawai-Yamada et al. 2004; Yoshinaga et al. 2005), (11) detection of ssDNA, (12) detection of calcium influx or its increase in cytosolic concentration, and (13) assessment of exposure of phosphatidylserine on the external side of a cell membrane (Palavan-Unsal et al. 2005).

Some of the above-presented ways to cell death determination, as it has been suggested by Kobori et al. (2007) and Ribble et al. (2005), have many limitations connected with the complicated procedure of detaching, washing, and transferring the studied material, leading to its partial loss; thus, they require more time and more material.

The aim of the studies presented in this paper was to prepare a simple digital method allowing determination of cell death *in planta*. This technique was based on the two most important typical features of dying cells, i.e., loss of cell membrane integrity and morphological changes in nuclei and nuclear chromatin. The loss of cell membrane integrity allows ethidium bromide (EB) to penetrate to the nuclei. Mixing EB and OA, it is possible to stain nuclei and show morphological changes in nuclear chromatin of dying cells. This method is frequently used to determine death-dependent features in animal cells (Azoulay-Zohar et al. 2004; van Doorn and Woltering 2005; Kobori et al. 2007; Palavan-Unsal et al. 2005; Ribble et al. 2005) and seems to have no limitations. To achieve the intended purpose, ethylene, which is a known inducer of cell death in plants (Drew et al. 2000; van Doorn and Woltering 2005; Vanyushin et al. 2002), and roots of *Vicia faba* ssp. *minor* seedlings were used.

## Material and methods

### Plant material, treatment, morphological analyses, and conductivity measurement

**Fig. 1:**
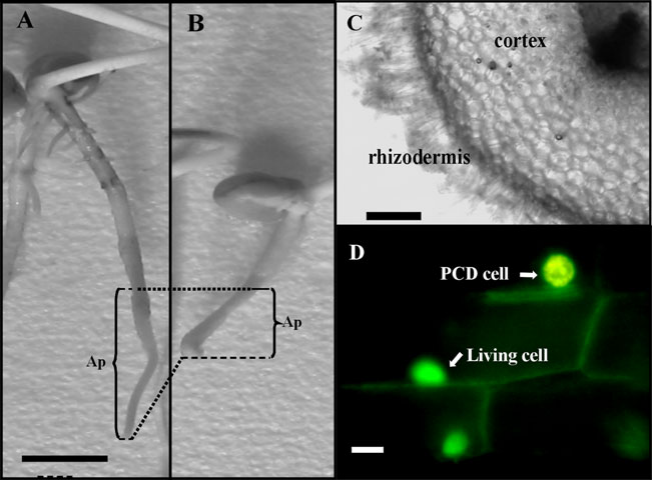
Control (**a**) and ACC-treated (**b**) seedlings of *V. faba* ssp. *minor* as well as microphotographs of cross sections of seedling roots showing cortex cells (**c**) with fluorescently (AO/EB) detected cell death (**d**). *Ap* apical part of roots. *Scale bars* are 20 mm (**a**), 100 μm (**c**), and 20 μm (**d**)

Seeds of *V. faba* ssp. *minor* were dark-germinated at 23°C in Petri dishes on blotting paper moistened with distilled water for 3 days and then transferred to a culture chamber and cultured with water (control) or with 1-aminocyclopropane-1-carboxylic acid (ACC, 5 μM) for the next 3 days. Then, seedlings were photographed, and the total length and width of roots were measured; next, 2-cm-long apical fragments of the control (Fig. 1a) and ACC-treated (Fig. 1b) roots were cut off and taken for analyses. The culture solution was used to measure conductivity with a conductivity meter (Elmetron, Poland).

### Microscopic fluorescence and morphological procedures to detect and estimate cell death

Detection of cell death in the root cortex cells of *V. faba* ssp. *minor* seedlings induced by ACC was carried out according to the following procedure: (1) the 2-cm-long apical fragments of roots were cut off with additional 3–4 mm to eliminate mechanically induced artifacts and immediately washed two times with 0.01 M phosphate buffer, pH 7.4 (PHB); (2) living fragments of roots were stained for 4 min with 1 ml of a “staining mixture” containing 100 μg ml^−1^ acridine orange (AO) and 100 μg ml^−1^ EB in PHB, (3) washed two times with PHB, (4) fixed with 1.0% gultardialdehyde (Merck) in PHB for 15 min, and (5) cut with a razor blade along the long axis of the root; (6) thin sections were washed two times for 2 min with PHB, put on glass slides with a drop of PHB, and analyzed using fluorescence microscopy with a blue filter (Kaźmierczak 2010).

Additionally, apical fragments of roots fixed in cold Carnoy's (96% ethanol and glacial acetic acid; 3:1) for 1 h, washed with 96% and 70% ethanol, and hydrated were stained with DAPI (Sigma) by 5-min pretreatment with 0.2 M citric acid and 0.1% Tween, 5-min staining with DAPI (2 μg ml^−1^) together with 0.1 M Na_2_HPO_4_ and 0.2 M citric acid in 9:1 ratio, and 5-min washing with the mixture of Na_2_HPO_4_ and citric acid. After this procedure, the roots were cut onto very thin sections, washed two times with PHB, and photographed under UV light of a fluorescence microscope (Kaźmierczak 2010). The microphotographs were used to present the morphological changes in nuclei and nuclear chromatin.

The morphological analyses of the root cortex were carried out with the *V. faba* ssp. *minor* seedlings fixed for 60 min with 2.5% gultardialdehyde in PHB and stained for 15 min at PHB with calcofluor white (Fluostain; Sigma). The cross sections were done with a razor blade, washed with PHB, analyzed, and photographed.

The Optiphot-2 epi-fluorescence microscope (Nikon) equipped with a camera and Act-1 software (Precoptic, Poland) was used for fluorescence and white light microscopy as well as microphotographs. The ScnImage software was used to measure the resultant fluorescence intensity (RFI) of AO and EB fluorochromes as well as surface and plot profile preparation.

### DNA extraction and separation

The 2-cm-long apical parts of roots were homogenized in 600 μl DNA extraction buffer (2% SDS; 0.5 M NaCl; 100 mM Tris–HCl, pH 8.0; and 50 mM EDTA, pH 8.0), using a plastic homogenizer and pestle, for 60 s. Homogenates were incubated, vortexing, at 65°C for 40 min, chilled on ice with 200 μl of 5 M potassium bi-sulphite for 10 min, and centrifuged (12,000×*g* for 10 min) at 4°C. Then, 1.0 volume of chloroform/isoamyl alcohol (24:1) was added to the samples, which were vigorously mixed by hand for 2 min and then centrifuged at 12,000×*g* for 1 min at 4°C. The supernatant was transferred to a fresh tube and extracted another time with 0.8 volume of cold isopropanol. The pellet was added with 300 μl of 70% ethanol, micro-centrifuged for 1 min (two times), dried, and re-suspended in TE buffer (1 mM Tris–HCl, pH 8.0, and 1 mM EDTA, pH 8.0) with 20 μg ml^−1^ RNase A. Separations of DNA samples and DNA marker (1 kb DNA Ladder, 250–10,000 bp; Ferementas) were run at 100 V for 3 h on a 1.5% (*w*/*v*) agarose gel with 0.50 μg ml^−1^ ethidium bromide. Separated DNA agarose gel was photographed under UV light with a Canon camera.

## Results

### Detection and measurement of ACC-induced cell death

**Fig. 2:**
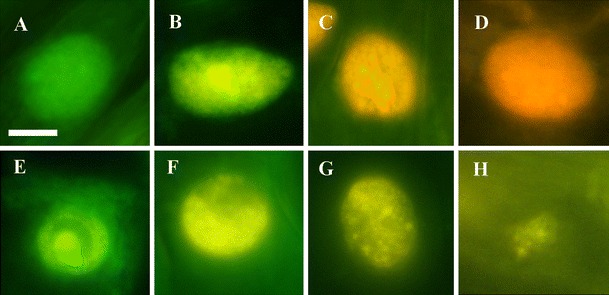
Micrographs of nuclei in living, PCD-dying, and dead cells in the root cortex of *V. faba* ssp. *minor* seedlings after ACC-induced PCD detected by AO/EB staining. *Green* nuclei of living cells (**a**), *green–yellow* nuclei of cells at early stages of PCD (**b**) with different forms of condensed (**e**, **f**) and disappearing (**g**, **h**) chromatin, *bright orange* nuclei of cells at the late stage of PCD (**c**) with condensed chromatin, and *bright red* nuclei of necrotic cells (**d**). The 10-μm *scale bar* in **a** is applied to all figures

**Fig. 3:**
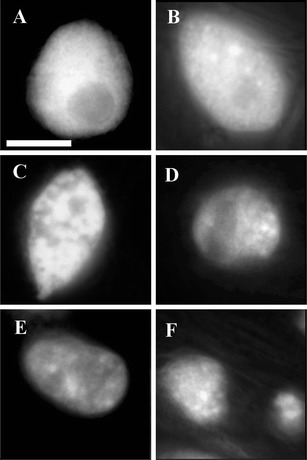
Micrographs of nuclei of ACC-treated *V. faba* ssp. *minor* seedling root cells fixed in Carnoy's and stained with DAPI showing the control nuclei (**a**), nuclei with condensation of chromatin (**b**, **c**), disappearance of nuclear chromatin (**d**, **e**), and nuclei fragmentation (**f**). The 10-μm *scale bar* in **a** is applied to all figures

Fluorescence microscopy with AO/EB staining revealed, in cortex cells (Fig. 1c) of ACC-treated roots of *V. faba* ssp. *minor* seedlings, differently stained nuclei (Figs. 1d, 2a–h), while nuclear chromatin showed various forms of condensation (Figs. 2a–d, 3a–c) and degradation (Figs. 2e–h, 3c, d) as well as nuclei fragmentation (Fig. 3e).

In the living cells, nuclei were green without condensation (Fig. 2a); in those dying, nuclei were green–yellow, yellow, yellow–orange, and bright orange (Fig. 2b, c, e–h), while in the dead cells, the nuclei were dark orange and bright red (Fig. 2c,d).

**Fig. 4:**
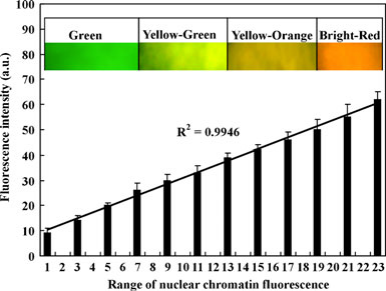
The curve of the resultant fluorescence intensity of nuclear chromatin stained with AO and EB used to distinguish living, dying, and dead cells and to determine the degree of damage in nuclei of root cortex cells during ACC-induced PCD in the *V. faba* ssp. *minor* seedlings

Measurement of AO/EB-stained nuclei showed that the RFI value increased when the color of the nuclei was changed from green to red. This fact was used to prepare the standard curve of the RFI (Fig. 4). In order to do it, the limits of green and red fluorescence emitted by the stained nuclear chromatin were assigned. Thus, the roots were separately stained with AO, EB, or AO/EB before or after fixation, and some were only stained. It turned out that green fluorescence was detected in the nuclei of both fixed and unfixed material after AO staining and in unfixed (living) material stained with the mixture of AO and EB. Dark orange and bright red fluorescence were emitted by the nuclear chromatin from the fixed material stained with AO/EB or EB. Measurement of the RFI of about 540–620 nuclei for each of the three experiments determined the value of fluorescence intensity of living and dead cells in the prepared standard curve.

According to that, nuclei of living cells fluoresced green (Fig. 2a) with fluorescence intensity between 9 and 26 arbitrary units (a.u.; Fig. 4) while uncondensed nuclear chromatin of dead cells fluoresced dark orange and bright red (Fig. 2d) with the intensity between 50 and 62 a.u. (Fig. 4). The green–yellow cells with or without condensed and fragmented nuclear chromatin (Fig. 2b, e–f) and yellow–orange and bright orange nuclei with condensed nuclear chromatin as well as disintegrated nuclei (Fig. 2c, g–h) revealed fluorescence between 27 and 49 a.u. (Fig. 4). Adopting apoptotic cell death models (Kobori et al. 2007), it was established that the green–yellow and yellow nuclei (Fig. 2b, e–f) with fluorescence between 27 and 38 a.u. (Fig. 4) were at the early stage of cell death while yellow–orange and bright orange (Fig. 2c, g–h) ones with fluorescence between 38 and 49 a.u. (Fig. 4) were at their late stage. Differences between fluorescence intensity of the nuclei in each type of cells were statistically important (*p* < 0.01).

**Fig. 5:**
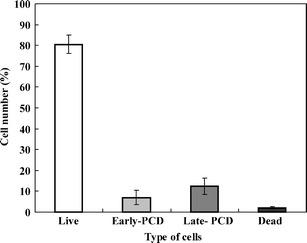
Index of living, dying (PCD), and dead cortex cells in roots of *V. faba* ssp. *minor* seedlings

These findings were used to distinguish and count living, dying, and dead cells in ACC-treated roots. It was revealed that 7% of cortex cells were at the early stage of death while 12.5% were at their late stage, 2% were dead, and the remaining ones were alive (Fig. 5). Differences between the numbers of cells were statistically important (*p* < 0.02)

### Effect of ACC on DNA degradation, aerenchyma formation, and on weight and length of roots and ion leakage

**Fig. 6:**
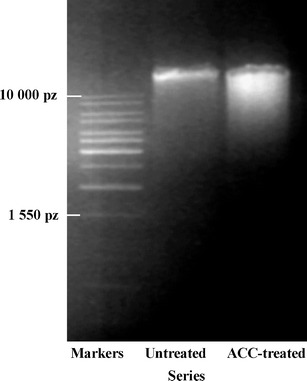
Electropherogram of DNA markers (1,550–10,000 bp) and samples of DNA isolated from the apical part of ACC-treated and untreated *V. faba* ssp. *minor* seedling roots

The agarose gel electrophoresis of DNA extracted from ACC-treated root showed small degradation, which was visible as a small “smear” (Fig. 6).

**Fig. 7:**
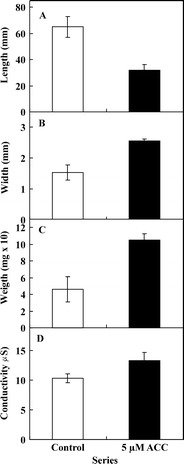
Length (**a**) and width (**b**) of whole roots as well as fresh weight of 2-cm-long apical parts of *V. faba* ssp. *minor* seedling roots (**c**) and conductivity of the culture solution (**d**) after ACC treatment

The ACC-induced cell death in *V. faba* ssp. *minor* seedlings was also accompanied by the reduction of length (from about 65 to 32 mm; Fig. 7a) and increment of width (from about 1.54 to 2.56 mm; Fig. 7b) of whole roots, increase in fresh weight (from about 4.6 to 10.5 mg) of 2-cm-long apical parts of roots (Fig. 7c) and increase (from about 10.4 to 13.4 μS; microsiemens) in the ion leakage secreted by the whole roots of seedlings measured by their conductivity (Fig. 7d).

**Fig. 8:**
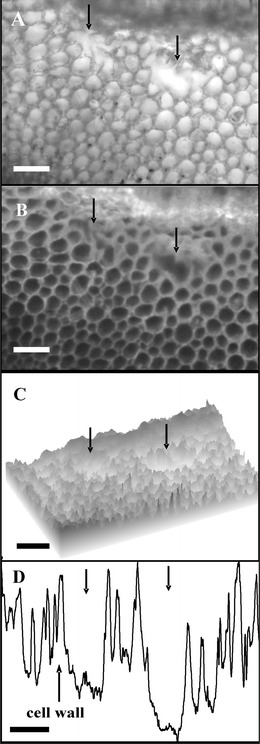
Cross section of the cortex of *V. faba* ssp. *minor* seedling roots with ACC-induced aerenchyma spaces (indicated by *arrows*), at the early stages of its formation, visualized under a *white light* (**a**), fluorescence, after cellulose with calcofluor white staining, (**b**) microscope, confirmed by plot profile (**c**) and surface plot (**d**). *Scale bars* are 100 μm

Moreover, morphological analyses of root cortex of *V. faba* ssp. *minor* seedlings under white light (Fig. 8a) and fluorescence (Fig. 8b) microscope showed forming aerenchymatic spaces. This fact was confirmed under surface (Fig. 8c) and profile (Fig. 8d) plots.

## Discussion

Some methods used to determine the cell death process created problems, especially when those which are routinely applied to study dying animal cells were applied to plant models (Jan et al. 2008; Palavan-Unsal et al. 2005).

DNA fluorescence staining with the mixture of acridine orange and ethidium bromide is one of such methods (Azoulay-Zohar et al. 2004; Kobori et al. 2007; Palavan-Unsal et al. 2005; Ribble et al. 2005; van Doorn and Woltering 2005). AO/EB staining consists in staining with AO which permeates whole cells and makes the nuclei green and EB which is only taken up by cells when the cellular membrane integrity is lost and stains the nuclei red. EB also dominates over AO (Ribble et al. 2005; Kobori et al. 2007). It has been reported that living cells have unchanged green nuclei; early apoptotic cells have bright green nuclei with slightly condensed or fragmented chromatin; late apoptotic cells display condensed and fragmented orange chromatin. Cells dying via necrosis have structurally normal orange nuclei (Ribble et al. 2005). Gohel et al. (1999) reported that in osteoblasts in which apoptosis was induced by glucocorticoids after AO/EB staining, nuclei of early apoptotic cells had yellow slightly condensed or fragmented chromatin. The late apoptotic cells had orange, condensed, and fragmented nuclear chromatin. Apoptotic cells also exhibited membrane blebbing while necrotic cells have bright orange chromatin in round nuclei (Gohel et al. 1999).

The above hints were used to adapt the AO/EB staining method to determine cell death in an *in planta* system. The well-known ethylene-dependent cell death model (Drew et al. 2000; Palavan-Unsal et al. 2005) with ACC, ethylene precursor was prepared with roots of *V. faba* ssp. *minor* seedlings. AO/EB staining showed differentiated fluorescence color of nuclei as well as condensation, degradation, and disappearance of nuclear chromatin only in the root cortex cells of ACC-treated plants. Condensation and degradation of nuclear chromatin, together with nuclei fragmentation, were also observed after DAPI staining. These results were in agreement with the data of Gohel et al. (1999) and Ribble et al. (2005) indicating that these hallmarks were typical not only of the animal but also of plant cell death processes (Cacas 2010; Jan et al. 2008; Palavan-Unsal et al. 2005; Ribble et al. 2005) also during hypoxia via ethylene lysigenous aerenchyma formation (Drew et al. 2000; van Doorn and Woltering 2005). It was indicated that ACC, the ethylene precursor, induced death of cortex cells in roots of *V. faba* ssp. *minor* seedlings. It allowed to prepare the standard curve of the RFI to determine the number of living, dying, and dead cells. It was noticed that about one fifth of cortex cells were dying, some of the cells were dead, and the rest of the cells were alive. Comparison of changes in color and morphology of nuclei of dying faba bean cells to those observed during apoptosis in animals (Ribble et al. 2005; Kobori et al. 2007) allowed to distinguish in the dying plant cells the early and late stages of the cell death process. The early stage of cell death with green–yellow and yellow nuclei with or without slight condensation of chromatin was observed while at the late stage, condensed and fragmented nuclei were with yellow–orange and bright orange chromatin, while the dark orange and bright red nuclei did not represent necrotic cells. Thus, they were described as dead.

It was reported that ethylene was involved in an aerenchyma formation (Drew et al. 2000; van Doorn and Woltering 2005) during which invagination of plasma membrane, more electron-dense cytoplasm and plasma membrane contraction from the cell wall, nuclear condensation, and formation of apoptotic bodies (Drew et al. 2000; Palavan-Unsal et al. 2005; van Doorn and Woltering 2005) and DNA ladder (Vanyushin et al. 2002) were observed. Some of these hallmarks (condensation of nuclear chromatin at the early and late stages and disintegration of nuclei at the late stage of cell death) were induced in the cortex cells of *V. faba* ssp. *minor* root seedlings. However, the apoptotic bodies were not noticed, while nuclei fragmentation was but not internucleosomal. It was probably connected with the fact that death of the root cortex cells was not synchronous because some cells were at the early and some at the late stage of cell death. It also indicated that ACC effect was transient, and in ACC-treated roots, the aerenchyma formation was observed, and after the next 2 or 3 days, the roots of ACC-treated seedlings looked like the control ones. Some authors (Drew et al. 2000; Vanyushin et al. 2002) suggest that the DNA ladder could not give the unequivocal information concerning ethylene-induced cell death These facts strongly confirm that ACC-induced PCD is a specific process, imitating ethylene action, inducing lysigenous aerenchyma formation (Drew et al. 2000).

It is known that ethylene is a plant growth hormone which leads to the inhibition of longitudinal expansion and promotes radial outgrowth of cells and organs (Buer et al. 2006; Dugardeyn et al. 2008). Similarly the ACC-induced cell death in the roots of *V. faba* ssp. *minor* seedlings was accompanied by shortening of whole roots and increment of width and weight of their apical part which was also accompanied by increase in the secreted cellular electrolyte conductivity, the hallmark which is frequently used to describe the programmed cell death process (Jan et al. 2008; van Doorn and Woltering 2005).

The results presented in this paper showed that ACC, like ethylene (Drew et al. 2000; van Doorn and Woltering 2005), induced PCD in root cortex cells of *V. faba* ssp. *minor*, of the non-wetland plant seedlings. Furthermore, measurements of the RFI with the use of appropriate software allowed to distinguish between living, dying, as well as dead cells while the value of florescence intensity according to the prepared standard curve allowed to count their number precisely, suggesting that the degree of cell membrane integrity loss was correlated with increasing RFI values. The above staining was previously applied to detect cell death during the development of *Anemia phyllitidis* fern gametophytes (Kaźmierczak 2008, 2010), and at present, AO/EB staining together with digital measurements was also successfully used to identify and measure cell death in the free-cell system of *Lycopersicon esculentum* (unpublished data).

Thus, the results presented in this paper and those obtained in unpublished studies showed that this simple, rapid, and accurate fluorescence procedure, which so far has been used only in plant and animal free-cell cultures (Gohel et al. 1999; Kobori et al. 2007; Ribble et al. 2005), might be applied as a complete method for routine determination of the PCD process in an *in planta* system. Moreover, changes in plasma and nuclear membranes as well as changes in morphology of nuclei are especially suitable to measure the cell death process because a cell membrane is the first feature of PCD (Jan et al. 2008), while the nucleus tends to be the last organellum to be degraded during cell death (van Doorn and Woltering 2005), and chromatin condensation as well as nuclei and DNA fragmentation are common hallmarks of cell death for plants and animals (Cacas 2010; Jan et al. 2008; Palavan-Unsal et al. 2005; van Doorn 2011). This is in agreement with the fact that neither apoptosis, which is not present in plants, nor necrosis, which should not be considered as an unprogrammed process (Cacas 2010; van Doorn et al. 2011), was induced by ACC in root cortex cells. Thus, ACC-induced cell death, imitating ethylene action, induced the autolytic kind of PCD process (Drew et al. 2000; van Doorn et al. 2011; van Doorn 2011; Vanyushin et al. 2002).
